# Antibiotic resistant airborne bacteria and their multidrug resistance pattern at University teaching referral Hospital in South Ethiopia

**DOI:** 10.1186/s12941-017-0204-2

**Published:** 2017-04-12

**Authors:** Fithamlak Bisetegen Solomon, Fiseha Wada Wadilo, Amsalu Amache Arota, Yishak Leka Abraham

**Affiliations:** School of Medicine, College of Health Sciences, Wolaita Sodo University, PO Box: 138, Wolaita Sodo, Ethiopia

**Keywords:** Airborne, Bacteria, Antibiotic, Resistance, Multi-drug resistance

## Abstract

**Background:**

Hospitals provide a reservoir of microorganisms, many of which are multi-resistant to antibiotics. Emergence of multi-drug resistant strains in a hospital environment, particularly in developing countries is an increasing problem to infection treatment. This study aims at assessing antibiotic resistant airborne bacterial isolates.

**Methods:**

A cross-sectional study was conducted at Wolaita Sodo university teaching and referral Hospital. Indoor air samples were collected by using passive air sampling method. Sample processing and antimicrobial susceptibility testing were done following standard bacteriological techniques. The data was analyzed using SPSS version 20.

**Results:**

Medically important bacterial pathogens, Coagulase negative *staphylococci* (29.6%), *Staphylococcus aureus* (26.3%), *Enterococci* species, *Enterococcus faecalis* and *Enterococcus faecium* (16.5%), *Acinetobacter* specie*s* (9.5%), *Escherichia coli* (5.8%) and *Pseudomonas aeruginosa* (5.3%) were isolated. Antibiotic resistance rate ranging from 7.5 to 87.5% was detected for all isolates. *Acinetobacter* species showed a high rate of resistance for trimethoprim-sulfamethoxazole, gentamicin (78.2%) and ciprofloxacin (82.6%), 28 (38.9%) of *S. aureus* isolates were meticillin resistant, and 7.5% *Enterococci* isolates of were vancomycin resistant. 75.3% of all bacterial pathogen were multi-drug resistant. Among them, 74.6% were gram positive and 84% were gram negative. Multi-drug resistance were observed among 84.6% of *P. aeruginosa*, of 82.5% *Enterococcii*, *E. coli* 78.6%, *S. aureus* 76.6%, and Coagulase negative *staphylococci* of 73.6%.

**Conclusions:**

Indoor environment of the hospital was contaminated with airborne microbiotas, which are common cause of post-surgical site infection in the study area. Bacterial isolates were highly resistant to commonly used antibiotics with high multi-drug resistance percentage. So air quality of hospital environment, in restricted settings deserves attention, and requires long-term surveillance to protect both patients and healthcare workers.

## Background

Hospital environment plays a significant role in the occurrence of nosocomial infection since it harbors a diverse population of microorganisms [1]. Bacterial pathogens of medical importance like *Pseudomonas aeruginosa*, *Staphylococcus aureus*, *Escherichia coli, Enterococci, Acinetobacter* spp. and Coagulase-negative *staphylococci* are a common cause of healthcare-associated infection which could able to survive and persist for long period of time in the hospital environment and have resistant disinfectants potential. The persistence ability of bacterial pathogens in hospital environment associated with a background rise in various types of nosocomial infections and could reach the sick patients through sources like air [2–5].

Operation theatre, delivery room, and intensive care unit are settings where patients are at a greater risk than the outside environment and could be polluted with bacterial pathogens released into it from various sources [6]. Environmental surface reservoirs like floors, the number of visitors, extent of indoor traffic, time of day and the number of materials brought in from outside and antibiotic resistance aggravate the extent of air bacterial microbiota [7, 8]. The uncontrolled movement of air in and out of the hospital environment makes the bacterial persistence worse since these infectious microorganisms may spread easily into the environment through sneezing, coughing, talking and contact with hospital materials. It can affect not only patients admitted to rooms in which the prior occupants tested positive for a pathogen but also other patients in the facility and even patients in other facilities in a network [9].

Hospitals provide a reservoir of microorganisms, many of which are multi-resistant to antibiotics. The emergence of resistance to antimicrobial agents is a global public health problem particularly in pathogens causing nosocomial infections which contributed to the morbidity, mortality, increased health care costs resulting from treatment failures, and longer hospital stays [1, 10].

The emergence of multi-drug resistant (MDR) strains in a hospital environment, particularly in developing countries is an increasing infection control problem presented a challenge in the provision of good quality patient care associated with high frequency of hospital acquired infections of which emergence, and reemergence of difficult-to-treat nosocomial infections in patients with increased antibiotic resistance rate [11, 12].

Frequently encountered MDR bacteria, methicillin-resistant *S. aureus*, cephalosporins, and extended spectrum beta-lactamase producing *Enterobacteriaceae*, ceftazidime-resistant *P.* aeruginosa, Imipenem-resistant *A. baumannii* and vancomycin-resistant *Enterococci* are commonly encountered in the hospital environment [13–15].

Post surgical site infection, urinary tract infection and respiratory infection are the common hospital acquired infection in this study area of which air could be the potential source. The susceptibility pattern of the isolates to commonly used antibiotics in this area will also provide enormous options for clinicians to select appropriate antibiotics for empirical therapy. So this study aims at the isolation and antibiotic susceptibility pattern of potentially pathogenic airborne bacteria in restricted settings of the hospital setup.

### Study area

The study was conducted at Wolaita Sodo University teaching and referral Hospital (WSUTRH), Sodo, located South Central Ethiopia. It’s serving people in catchment’s area of 2 million people. The hospital has 320 beds for inpatient service which are on medical, pediatrics, surgical, gynecology and obstetrics ward.

### Study design

The Hospital-based cross-sectional study design was conducted in WSUTRH from November 2015–March 2015.

### Sample size

Sample number were determined in convenience in which 72 settle plate samples were collected in each ward (Delivery room, operation theater, and intensive care unit) for continuous 3 months which gives a total sample size of 216 airborne samples.

### Sampling techniques

The air samples were collected from November 1–March 30, 2015 (1st week of the months) once a week during Monday’s since the patient load is higher in the study area. The air samples were collected in a day considering the most representative hours (at 8–9 AM, at 11 AM–12 PM, 4–5 PM) after a preliminary survey by considering the fact that a higher patient, staff and attendee load could become the highest burden for acquiring infection through air way. Indoor air samples were collected from delivery room, intensive care unit and operation theatre. Settle plate or passive air sampling method was used. A nine cm diameter sterile Petri dish with 20 ml tryptic soy agar (TSA) was left open to the air for an hour, a meter above the floor and a meter from the wall [16]. Self-contamination was prevented by wearing sterile surgical gloves, mouth masks, and protective gown. Petri-plates were labeled with sample number, hospital ward, date and time of sample collection. Two agar plates were placed at each of the selected wards with 5 m apart. Soon after collection; samples were transported to the microbiology laboratory in sealed plastic bags.

### Processing of specimens and preliminary identification

Following collection, 3–5 colonies on TSA were inoculated into MacConkey agar, blood agar plates 5% (BAP), brain heart infusion agar, Mannitol salt agar (Oxoid, LTD), and Bile-aesculin-azide agar (BEAA) (Biomerieux, France) selective medium. The inoculated agar plate was incubated at 35 °C for 24–48 h. Then the growth was inspected to identify the bacteria. Microbial growth on the agar media was identified by colonial morphology, Gram staining, and biochemical tests, oxidase, catalase, coagulase, citrate, indole test, growth in 6.5% NaCl and turbidity, voges-prosquaer, hippurate hydrolysis, pigment production and mannitol fermentation. Isolates were determined and characterized based on Bergy manual of determinative bacteriology [17].

### Antibiotic susceptibility testing

Susceptibility testing was performed on isolates based on the Kirby–Bauer diffusion technique [18]. The grades of susceptibility pattern were recognized as sensitive, intermediate and resistant by comparison of the zone of inhibition as indicated in Clinical lab science institute [19] standard 2014. The antibiotics tested for both gram negative and gram positive bacteria (Oxoid, Basingtone, UK) were amikacin (30 μg), ampicillin (10 μg), amoxicillin (25 μg), amoxicillin-clavulanic acid (30 μg), cefoxitin (30 μg), ceftazidime (30 μg), ceftriaxone (30 μg), chloramphenicol (30 μg), ciprofloxacin (5 µg), clindamycin (2 µg), doxycycline (30 μg), erythromycin (15 µg), gentamicin (10 μg), imipenem (10 μg), norfloxacin (10 μg), penicillin G (10 units), tetracycline (30 μg), trimethoprim-sulphamethoxazole (25 μg), and vancomycin (30 µg). Antibiotics were selected based on local availability, literature, effectiveness and CLSI Guidelines.

MDR was defined as acquired non-susceptibility to at least one agent in three or more antimicrobial categories [20].

Pan resistance-Resistance for all antibiotics tested.

### Quality controls

Standard operating procedures were prepared and followed from sample collection to reporting. Culture medias was prepared based on the manufactures instruction then the sterility of culture media was checked by incubating 5% of the batch at 35–37 °C overnight and observing bacterial growth. Those culture medias which showed growth were discarded. Antibiotic discs potency was checked by using *S. aureus* ATCC25923, *E. coli* ATCC 25922 and *P. aeruginosa* ATCC 27853 strains as control organisms.

### Data analysis

Statistical analysis was performed by using SPSS version 20 software program and descriptive statistics were used.

### Ethical considerations

The proposal was approved by the ethical review committee of Wolaita Sodo University. An official letter was written from the university to WSUTRH administrator. The result of the study was communicated to the responsible bodies for any beneficiary or corrective measures.

## Results

**Fig. 1:**
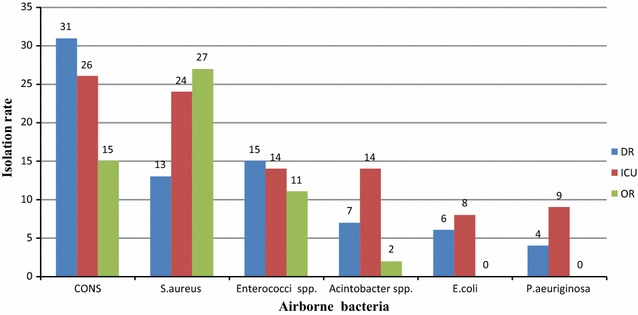
Distribution of airborne bacterial pathogens in wards of WSUTRH, November–March 2015. *DR* delivery room, *ICU* intensive care unit, *OR* operation theater

Total numbers of 216 air samples were collected from delivery room (DR), intensive care unit (ICU), and operation theatre (OT). 195 (90.2%) settle plates were showed a positive bacterial growth for any one of the bacteria. Significant proportions of bacterial pathogens of nosocomial importance like Coagulase negative *staphylococci*, *S. aureus, Enterococci* spp., *E. fecalis*, 72.5% and *E. faecium*, 27.5%, *Acinetobacter* spp., *E. coli*, and *P. aeruginosa* were also detected. CoNS (72/243) were the highest prevalent bacteria in three wards followed by *S. aureus* 45.8% (64/243). Coagulase negative *staphylococci* 37.8% were the most accountable bacteria in the delivery room followed by *S. aureus* 42.2% (27/64). On the other hand, *S. aureus* was the most abundant bacteria identified in the OR 27/59 (45.85). CoNS and *S. aureus* was the most isolated bacteria in the ICU Fig. 1.

### Antibiotic resistance rate of air-borne bacterial pathogens

Antibiotic resistance range of 7.5 up to 87.5% was detected among gram-positive isolates of which CoNS showed a high level of resistance for chloramphenicol 68.1% and trimethoprim-sulfamethoxazole 66.7%. *S. aureus* isolates depicted 87.5% resistance to chloramphenicol and 28 (38.9%) were MRSA isolates. *Enterococci* spp. showed resistant for trimethoprim-sulfamethoxazole 77.5% and ampicillin, 70%. Eight isolates in this study were vancomycin-resistant *Enterococci* (VRE).

Gram-negative bacterial isolates showed resistance ranging from 15.4–87% of which *Acinetobacter* spp. showed a high rate of resistance (>65%) for all antibiotics tested. Ampicillin and doxycycline resistant *E. coli* isolates 71.4 and 78.6% respectively were identified. Imipenem, ceftazidime, and ciprofloxacin resistance *P. aeruginosa* isolates with the rate of resistance, 38.5, 23.1, and 76.9% were also isolated respectively (Table 1).

**Table 1:** Antimicrobial resistance pattern of airborne bacterial pathogens of nosocomial importance in WSUTRH, November–March 2015

Resistance						
Gram positive				Gram negative		
Antibiotics	CoNS (72)	*S. aureus* (64)	*Entrococci* spp. (40)	*Acinetobacter* spp. (23)	*E. coli* (14)	*P. aeruginosa* (13)
AMP	ND	ND	28 (70)	ND	10 (71.4)	ND
AMK	ND	ND	11 (27.5)	16 (69.6)	2 (15.4)	6 (46.2)
AMX-CLA	ND	ND	ND	ND	5 (35.7)	ND
AZT	20 (27.8)	17 (26.6)	13 (32.5)	ND	ND	ND
CAF	49 (68.1)	56 (87.5)	25 (62.5)	ND	ND	ND
CF	24 (32.8)	28 (38.9)	ND	ND	ND	ND
CFZ	ND	ND	ND	15 (65.2)	ND	3 (23.1)
CIP	23 (31.9)	25(39.1)	23 (57.5)	19 (82.6)	7 (50)	8 (61.5)
CN	35 (48.6)	21 (32.8)	12 (30)	18 (78.2)	2 (15.4)	8 (61.5)
CRO	ND	ND	14 (35)	18 (78.2)	5 (35.6)	8 (61.5)
CPH	ND	ND	27 (67.5)	ND	ND	ND
DA	12 (16.7)	16 (25)	16 (40)	ND	ND	ND
DOX	ND	ND	ND	20 (87)	10 (71.4)	ND
ERT	ND	ND	ND	ND	ND	ND
IMP	ND	ND	ND	ND	7 (50)	5 (38.5)
NOR	ND	ND	ND	ND	ND	9 (69.2)
PEN	ND	ND	30 (75)	ND	ND	ND
TMP-SXT	48 (66.7)	35 (54.7)	31 (77.5)	19 (82.6)	5 (35.7)	9 (69.2)
VAN	ND	ND	3 (7.5)	ND	ND	ND

*Amk* amikacin, *Amp* ampicillin, *Amx* amoxicillin, *Amo-cla* amoxicillin-clavulinic acid, *CF* cefoxitin, *Cro* ceftriaxone, *CFZ* ceftazidime, *Cph* cephalothin, *Caf* chloramphenicol, *Cip* ciprofloxacin, *DA* clindamycin, *DOX* doxycycline, *Ert* erythromycin, *Cn* gentamicine, *IMP* imipenem, *Nor* norfloxacin, *PEN* penicillin, *Tmp-Sxt* trimethoprim-sulfamethoxazole, *VAN* vancomycin, *ND* not done

### MDR pattern of gram-positive airborne bacterial pathogens

Seventy-five percent of all bacterial pathogens were multi-drug resistant (MDR) of which 74.6% were gram positive and 84% were gram negative. *Enterococci* spp. accounts 82.5% MDR prevalence of which three isolates were pan-resistant and seven isolates were resistant for three antibiotics (chloramphenicol + gentamicin + trimethoprim-sulfamethoxazole. Seventy-three (73.6%) CoNS became MDR of which two isolates depicted pan-resistance. *S. aureus* isolates showed the very high level of MDR rate, 76.6% (Table 2).

**Table 2:** Multi drug resistant pattern of airborne gram positive bacterial isolates in WSUTRH November–March 2015

Bacteria	Quantity	Antibiotics pattern	Frequency	Class
*Enterococci* spp.	Max	AMK, AMP, AZT, CAF, CIP, CN, CPH, CRO, DA, PEN, TMP-SXT, VAN	3	9
	Min	AMK, TMP-SXT, PEN	2	3
		CAF, CN, TMP-SXT	7	3
		DA, PEN, TMP-SXT	2	3
CoNS	Max	AZT, CAF, CF, CIP, CN, DA, DOX, TMP-SXT	2	8
	Min	CAF, DOX, TMP-SXT	8	3
		AZT, CAF, TMP-SXT	2	3
		CF, CN, TMP-SXT	6	3
		DOX, CIP, TMP-SXT	8	3
*S. aureus*	Max	AZT, CAF, CF, CIP, CN, DA, DOX, TMP-SXT	4	8
	Min	CF, DA, TMP-SXT	7	3
		AZT, CAF, DOX	8	3
		CN, CIP, TMP-SXT	8	3

*Max* maximum number of resistance pattern, *Min* minimum number of resistance pattern

### MDR pattern of gram-negative airborne bacterial pathogens

Overall MDR rate of gram negative bacteria were 84%. The vast majorities (91.3%) of *Acinetobacter* spp. were MDR with two pan-resistant isolates and 84.6% of the isolates, four pan-resistances, were MDR. Eighty percent of *E. coli* isolates were having multi-drug resistance of which 11.1% of the isolates depicted MDR pattern of ampicillin + amoxicillin-clavulanic acid + cephalothin + ciprofloxacin + trimethoprim-sulfamethoxazole) (Table 3).

**Table 3:** Multi-drug resistance pattern of airborne gram negative bacterial pathogens in WSUTRH from November–March 2015

Bacteria	Quantity	Antibiotic pattern	No	Class
*P. aeruginosa*	Max	AMK, CFZ, CIP, CN, CRO, IMP, NOR, TMP-SXT	2	5
		AZT, CFZ, CIP, CN, IMP, NOR	1	
		AMK, CIP, CN, NOR, TMP-SXT	2	
	Min	AMK, CIP, CN, CRO	2	3
*Acinetobacter* spp.	Max	AMK, CFZ, CIP, CN, CRO, DOX, TMP-SXT	4	5
		AMK, CFZ, CIP, CN, DOX, TMP-SXT	3	
		AMK, CFZ, CIP, CRO, DOX, TMP-SXT	4	
		CIP, CN, CRO, DOX, TMP-SXT	3	
	Min	CFZ, CN, CRO, DOX	4	3
*E. coli*	Max	AMK, AMP, AM-CLA, CN, CRO, DOX, TMP-SXT	2	6
	Min	AMP, CIP, DOX, IMP	3	4
		AMP, DOX, IMP, TMP-SXT	3	

## Discussion

CoNS, and *S. aureus*, identified in this study is in line with previous findings conducted in, Ethiopia [21, 22], Nigeria [23], Kashmir [24], Nepal [25], and Iran [26]. CoNS and *S. aureus* were the predominant bacterial isolates in all setups in this study which could be associated with their ability to persist and resist the harsh environmental condition and to suspend in the air particles [9]. *S. aureus*, was the most prevalent bacteria in the air of operation theatre. This finding is corroborated with findings done previously which has the same [21–23, 26]. This might be because of its survival ability on the environmental surface, ability to resist disinfection and could also have an association with the fact that *S. aureus* was the main cause of post-surgical site infection in this hospital.

*Enterococci* spp. (*E. fecalis* and *E. faecium*) reported in this study were also identified in previous studies isolated elsewhere in Nigeria [23] and Iran [27]. Whereas *Acinetobacter* spp. 9.3% was the most common gram-negative bacteria in this study which could be supported by its higher survival ability (3 days to 11 months) in the environment. Our finding couldn’t be compared with other airborne studies in Ethiopia since it is not reported even though these bacteria were reported elsewhere, Taiwan 13.4% [28] and Iran 42% [26] in restrict settings like ICU and OT. *Enterococci* and *Acinetobacter* spp. did not reported previously from air studies in Ethiopia. This could possibly be due to many reasons, the investigator’s attention to these pathogens, requirement of high sterility aseptic techniques with enriched bacterial media (selective) and reporting of these bacteria in family name (*streptococci*) rather than species name.

The isolation percentage of *E. coli* (7.5%) in this study is lower as compared with the 13.5% detected in Khartoum [29] hospital and 15.2% reported in Taiwan hospital [28]. This could be due to the use of active air sampling in Taiwan hospital and difference in ward and number of patients attended in reference to Khartoum hospital as compared and *E. coli* is one of the commonest causes of urinary tract infections, 35% prevalence in the study setting where *E. coli* accounts 31.4%, and is commonly present in appendix abscess, peritonitis, cholecystitis, septic wounds and bedsores, bacteraemia and endotoxic shock, particularly in surgical or otherwise debilitated patients [30].

*Pseudomonas aeruginosa* prevalence (5.3%) in this study is relatively comparable with other similar study in North Ethiopia [22] but much less than 28% [31] and 52% [10] reported previously which could be due to the difference in methodology, sampling place or magnitude of nosocomial infection in specific localities. *P. aeruginosa* associated infection is a recognized public health threat often acquired from the hospital environment and contaminated medical devices. It is not only an important cause of morbidity but also increases the stay of the patient in the hospital and increases the cost of treatment [32].

Indoor air quality in a hospital environment is of great concern to patients, attendants, and clinical staff which could be a cause for nosocomial infections and outbreak. High level of airborne bacteria in the hospital is an alarming call since these settings are places where actual surgical procedures, severely sick, and post-surgical rehabilitated patients are admitted and delivery services are handled.

Antibiotic resistant infections add considerable and avoidable costs to the health care system of which it adds 20 billion USD in excess direct health care cost with additional costs to low society of productivity as high as 35 billion USD [33].

Thirty-nine percent of MRSA prevalence in the current study is higher than 26.6% [34], 18.1% [35] and 7.7% [22] reported in Ethiopia which could be explained by difference in sampling site where most of the resistant isolates in the current study were identified in the intensive care unit where repeated exposure to antibiotics is administered. The frequency of antibiotic administration and magnitude of nosocomial infection in each locality also may differ. Emergence of drug resistant strains especially methicillin-resistant *S. aureus* is a serious problem in hospital environments and infections caused by MRSA strains, 51% prevalence (hospital lab record for 8 years) are associated with longer hospital stay, prolonged antibiotic administration, and higher cost than infections caused by methicillin-susceptible *S. aureus* strains [36].high prevalence of MRSA strains from the environment, 39% could become the main factors for 51% resistance rate from clinical isolates.

Seventy 70 and 75% resistance prevalence for ampicillin and penicillin G *Enterococci* spp. in this study has a great implication since these antibiotics are preferable in the treatment of enterococcal infections [37] and the growing incidence of infections is a great concern due to multidrug resistance. Vancomycin-resistant *Enterococci* (VRE) 9.1% prevalence in the current study make the scenario worse since vancomycin is the preferred choice in the case where this bacteria became resistant to other antibiotics. CoNS and *S. aureus* also showed a high level of resistance for chloramphenicol and trimethoprim-sulfamethoxazole.

Gram-negative bacteria showed a high level of MDR rate than gram-positive bacteria in the current study that *Acinetobacter* spp. and *P. aeruginosa* showed high resistance rate for most of the antibiotics. *Acinetobacter* spp. showed the highest rate of resistance with a minimum resistance of 61.5%, this could possibly be due to the bacterial ability to resist many antibiotics or could possibly be due to selective pressure or abusing of the drugs in the hospital where 86.6% prevalence were recorded for commonest antibiotics like ciprofloxacin, and gentamicin. A higher number of resistances to effective antibiotics like ceftazidime 23.1% and imipenem 38.5% to *Acinetobacter* are a major public health problem since their disclosure could possibly lead to a therapeutic impasse in the hospital.

Higher levels of *P. aeruginosa* resistance were noticed for TMP-SXT 69.2%, gentamicin 61.5% and ceftriaxone 61.5% which is comparable with the study conducted in Ethiopia [31] of which 95, 62.5, and 58.2% resistance were detected on TMP-SXT, gentamicin and ceftriaxone antibiotics respectively.

## Conclusions and recommendation

Higher prevalence airborne bacteria of clinical concern, CoNS, *S. aureus, Enterococci* spp., *Acinetobacter* spp., *P. aeruginosa* and *E. coli* were found in WSUTRH. This could be significant factors for the high prevalence of post-surgical site infection and respiratory tract infection in the study area. High MDR resistance rate (>70%) of the isolates on the hospital air is a clinical concern since antibiotic resistant rate increased in alarming rate. Effective and new antibiotics, like ceftazidime and imipenem resistance, for the study area in the hospital air could be projected to the patients and could pose major problem for antibiotic stewardship programs. So this findings deserves attention, and requires effective infection control measures like proper disinfection and regular cleaning, restriction of patient relatives’ movement in and out of the wards/units to protect both patients and healthcare workers. Though isolates were not identified from patients in this study, the role of contaminated indoor air could be pathogenic if contact is established with patients; it is pertinent that their presence should be controlled.

## Abbreviations

DR: delivery room
ICU: intensive care unit
MDR: multi drug resistant
OT: operation theatre
TMP-SXT: trimethoprim-sulfamethoxazole
WSUTRH: Wolaita Sodo University teaching referral Hospital
